# Correlation of Clinical Fibrillar Layer Detection and Corneal Thickness in Advanced Fuchs Endothelial Corneal Dystrophy

**DOI:** 10.3390/jcm11102815

**Published:** 2022-05-17

**Authors:** Orlando Özer, Mert Mestanoglu, Antonia Howaldt, Thomas Clahsen, Petra Schiller, Sebastian Siebelmann, Niklas Reinking, Claus Cursiefen, Björn Bachmann, Mario Matthaei

**Affiliations:** 1 Department of Ophthalmology, Faculty of Medicine and University Hospital Cologne, University of Cologne, 50937 Cologne, Germany; orlando.oezer@uk-koeln.de (O.Ö.); mert.mestanoglu@uk-koeln.de (M.M.); antonia.howaldt@uk-koeln.de (A.H.); thomas.clahsen@uk-koeln.de (T.C.); sebastian.siebelmann@uk-koeln.de (S.S.); niklas.loreck@uk-koeln.de (N.R.); claus.cursiefen@uk-koeln.de (C.C.); bjoern.bachmann@uk-koeln.de (B.B.); 2 Eye Center Seufert, 51427 Bergisch Gladbach, Germany; 3 Center for Molecular Medicine Cologne (CMMC), Faculty of Medicine and University Hospital Cologne, University of Cologne, 50937 Cologne, Germany; 4 Institute for Medical Statistics and Bioinformatics, Faculty of Medicine and University Hospital Cologne, University of Cologne, 50937 Cologne, Germany; petra.schiller@uk-koeln.de

**Keywords:** Fuchs endothelial corneal dystrophy, fibrillar layer, Scheimpflug imaging, backscatter, densitometry, corneal thickness, pachymetry

## Abstract

Central subendothelial geographic deposits are formed as a fibrillar layer (FL) in advanced Fuchs endothelial corneal dystrophy (FECD). Previous studies demonstrated a significant decrease in corneal endothelial cell (CEC) density and an increase in focal corneal backscatter in the FL area. The present study investigated the association of the FL with edema formation and its localization. Patients (*n* = 96) presenting for Descemet membrane endothelial keratoplasty (DMEK) for advanced FECD were included. Slit-lamp biomicroscopy with FECD grading was followed by Scheimpflug imaging with en face backscatter analysis and pachymetric analysis. FL dimensions were measured, and correlation with pachymetric values was performed. An FL was detected in 74% of all eyes (*n* = 71). Pachymetric values in FL-positive versus FL-negative eyes were for corneal thickness at the apex (ACT) 614 ± 52 µm and 575 ± 46 µm (*p* = 0.001), for peripheral corneal thickness at 1 mm (PCT_1mm_) 616 ± 50 µm and 580 ± 44 µm (*p* = 0.002), for PCT_2mm_ 625 ± 48 µm and 599 ± 41 µm (*p* = 0.017), for PCT_3mm_ 651 ± 46 µm and 635 ± 40 µm (*p* = 0.128) and for PCT_4mm_ 695 ± 52 µm and 686 ± 43 µm (*p* = 0.435), respectively. Correlation analysis indicated a weak correlation for the FL maximum vertical caliper diameter with ACT and PCT_1mm_ values but no further relevant correlations. In FL-positive eyes, increased focal corneal backscatter and increased corneal thickness showed primarily central and inferotemporal localization. In conclusion, Scheimpflug imaging shows an association of the FL with increased corneal thickness in advanced FECD and shows localization of the FL and increased corneal thickness in the central and inferotemporal region. This may provide important information for progression assessment and therapeutic decision making in FECD patients in the future.

## 1. Introduction

Fuchs endothelial corneal dystrophy (FECD) is a bilateral disease of the corneal endothelium. It represents the most common indication for corneal transplant surgery and accounts for approximately 39% of the total number of keratoplasties performed worldwide [1,2].

The pathomorphology of FECD is characterized by an accelerated loss of corneal endothelial cells and subendothelial accumulation of altered extracellular matrix with the formation of posterior excrescences (guttae) of Descemet’s membrane [2]. Decreased endothelial cell density results in impaired pump function and an increased leakage of aqueous humor into the stromal tissue with subsequent corneal edema formation and scarification in the long-standing disease [2]. Symptomatic visual limitations result from altered refraction, optical aberrations and light scattering [3,4,5,6].

Since the initial description of FECD in 1910 [7], numerous classification systems have been developed to document the progression of the disease based on its characteristic pathomorphology but also based on the changes in the optical properties of the cornea and the subjective impairment of FECD patients. The Krachmer grading is the most widely applied classification system and uses slit-lamp biomicroscopy detection of guttae distribution and of clinical corneal edema formation as the main progression criteria [8].

The advancement of new imaging techniques facilitates the stage-specific identification of pathomorphologic alterations in FECD in even greater detail. This allows for a refined assessment of disease progression also fostering the process of reliable surgical decision making.

In this context, our previous studies demonstrated by correlation of FECD histology and clinical imaging that collagen-rich deposits form as a fibrillar layer (FL) between the central endothelium and guttae of Descemet’s membrane in approximately 80% of advanced FECD eyes [9,10]. This FL represents a significant source of corneal backscatter and may be visualized by en face Scheimpflug backscatter imaging in vivo. Moreover, it was shown that the FL marks areas of significant decrease in corneal endothelial cell (CEC) density [9,10].

The present study further investigated whether this marked decrease in endothelial cell density in the FL area is accompanied by increased edema formation. Scheimpflug en face backscatter imaging and tomography was applied to analyze the FL localization and the association between clinical FL detection and alterations in corneal pachymetry as a sign of focal edema formation in vivo.

## 2. Materials and Methods

### 2.1. Study Design and Patient Selection

In a retrospective analysis of prospectively collected data, consecutive patients presenting for Descemet membrane endothelial keratoplasty (DMEK) for advanced FECD at the Department for Ophthalmology, University Hospital Cologne, Germany, in the period from October 2020 to April 2021 were included. Inclusion criteria were the diagnosis of advanced stage FECD (modified Krachmer grade 5 or 6) [8,11] confirmed by slit-lamp biomicroscopy, the existence of a preoperative high-quality Scheimpflug image (Pentacam HR, Oculus GmbH, Wetzlar, Germany; software version 1.22r09, software Quality Status: ok) acquired no more than six months before surgery and an age ≥18 years on the day of consent and inclusion. Exclusion criteria were the presence of corneal diseases other than FECD and/or previous corneal surgery in the study eye. In addition, patients who did not agree or were unable to provide written informed consent to the study were not included. Formal approval to conduct this study was obtained from the Ethics Committee of the University of Cologne (16-424). Written informed consent has been obtained from all participants for the treatment and participation in the research. The research adhered to the tenets of the Declaration of Helsinki.

### 2.2. Clinical Examination and Imaging

Slit-lamp biomicroscopy with confirmation of FECD diagnosis and modified Krachmer grading was performed by one of four corneal specialists (OO, CC, BB, MMa): Grade 1: 1–12 non-confluent central guttae; Grade 2: >12 non-confluent central guttae; Grade 3: confluent guttae over 1–2 mm in diameter; Grade 4: confluent guttae over 2–5 mm in diameter; Grade 5: >5 mm diameter confluent guttae; Grade 6: >5 mm diameter confluent guttae and corneal edema [8,11]. Scheimpflug imaging (Pentacam, Oculus GmbH) was performed as part of the routine diagnostic workup, including en face Scheimpflug backscatter analysis (Densitometry display, Pentacam software version 1.22r09) and Scheimpflug pachymetry analysis (Pachymetry display, Pentacam software version 1.22r09) [10]. Because previous studies have demonstrated the diurnal dependence of pachymetry values in advanced FECD [3,12], the time of day of Pentacam imaging was recorded as AM or PM.

#### 2.2.1. Scheimpflug Imaging

Patients were placed in front of the Pentacam (Oculus GmbH), and the head was fixed to the chin rest and forehead strap. Gaze fixation was performed by an optical fixation object. All images were acquired under unchanged room light conditions. The light source of the Pentacam (Oculus GmbH) is a blue light-emitting diode (LED) with a wavelength of 475 nm. Combined with slit-shaped illumination, a radially oriented rotating measurement procedure generates Scheimpflug cross-sectional images of the anterior segment of the eye. After initialization, 25 images are automatically generated in less than 2 s.

**Figure 1:**
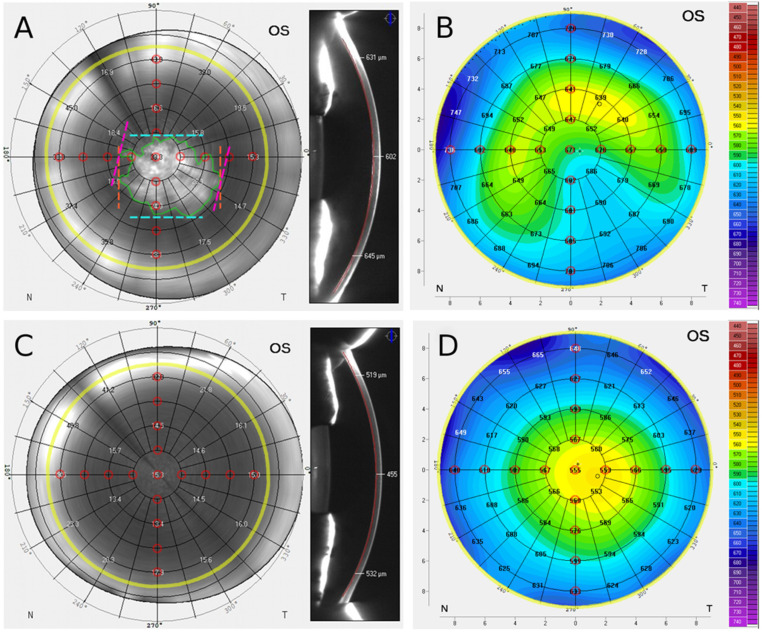
Scheimpflug backscatter and pachymetry in fibrillar layer (FL)-positive (**A**,**B**) and FL-negative (**C**,**D**) Fuchs endothelial corneal dystrophy (FECD) eyes: (**A**) En face Scheimpflug backscatter image of FL-positive FECD cornea: horizontal caliper diameter (orange), vertical caliper diameter (blue), maximum caliper diameter (pink) and FL area (green). FL shows inferotemporal localization. (**B**) Pachymetry map of FL-positive cornea: Increased corneal thickness inferotemporal. (**C**) En face Scheimpflug backscatter image of FL-negative cornea, (**D**) Pachymetry map of FL-negative cornea; superimposed grid marks concentric annuli around the corneal apex at 1 mm radial spacing (black) and at 4.5 mm radius (yellow). The study analyzed backscatter and pachymetry values along the vertical and horizontal axes (red circular marks).

Scheimpflug imaging data were analyzed by a single cornea specialist (OO) following instruction and joint grading of a pilot series with another experienced grader and cornea specialist (MMa). In the Densitometry display (Pentacam software version 1.22r09), the en face Scheimpflug backscatter plane with the highest backscatter gray scale unit (GSU) at the corneal apex within the posterior corneal 100 µm was selected as described before [10]. The image was exported in high-resolution quality. Concentric annuli around the corneal apex superimposed by the Pentacam software served as a metric scale reference (Figure 1). The presence of an FL was defined as a central or paracentral geographic hyperreflectivity with a maximum caliper diameter >1 mm [10]. The boundaries of the FL were manually marked using an image analysis software (ImageJ Version 1.53e, NIH, USA, Figure 1), and the FL area was calculated [13,14]. In addition, manual marking of the horizontal and the vertical caliper diameters of the FL was performed. These caliper diameters are defined as the longest distance between two points on the FL along the x and y axes, respectively (Figure 1). The maximum caliper diameter of the FL was automatically calculated by the image analysis software (Figure 1) [10]. 

The focal apex corneal backscatter (ACB) value was extracted, and the focal peripheral corneal backscatter (PCB) values were extracted at a radius of 1 mm (PCB_1mm_), 2 mm (PCB_2mm_), 3 mm (PCB_3mm_) and 4 mm (PCB_4mm_) from the apex along the vertical and horizontal axes (Figure 1). Radius-specific focal PCB values were determined by calculating the mean of the four focal backscatter values located in the respective superior, nasal, inferior, and temporal position (Figure 1).

#### 2.2.2. Scheimpflug Pachymetry Data

The pachymetry map was exported from the Pentacam pachymetry display in high-resolution quality. The central corneal thickness (CCT) and apex corneal thickness (ACT) values were extracted and the peripheral corneal thickness (PCT) values were extracted at a radius of 1 mm (PCT_1mm_), 2 mm (PCT_2mm_), 3 mm (PCT_3mm_) and 4 mm (PCT_4mm_) from the apex along the vertical and horizontal axes (Figure 1). The radius-specific PCT values were determined by calculating the mean of the four pachymetry values located in the superior, nasal, inferior, and temporal location, respectively (Figure 1).

#### 2.2.3. Comparison of Fibrillar Layer-Positive and -Negative Eyes 

Patients were divided into an FL-positive group and an FL-negative group. Scheimpflug backscatter data and pachymetry data were compared between both groups. 

In order to analyze the correspondence of the FL localization and of areas of increased corneal thickness, individual focal backscatter values (en face backscatter plane at the level with the highest backscatter gray scale unit (GSU) at the corneal apex within the posterior corneal 100 µm as described above) and pachymetry values were compared between FL-positive and FL-negative corneas at a total of 17 positions within the concentric annuli grid around the corneal apex superimposed by the Pentacam software (corneal apex as well as superior, nasal, inferior, temporal location at 1 mm, 2 mm, 3 mm and 4 mm radial distance from the apex, Figure 1). Comparing FL-positive to FL-negative eyes, significantly elevated focal GSU levels were regarded as indicative of increased presence of an FL, and significantly elevated pachymetry values were regarded as indicative of increased presence of corneal edema in the respective cohort.

#### 2.2.4. Correlation Analysis 

Correlation of fibrillar layer dimensions and pachymetry: A correlation analysis was performed in FL-positive eyes to determine correlation between FL dimension parameters (FL area, vertical and horizontal caliper diameters, maximum caliper diameter) and pachymetric parameters (CCT, ACT, PCT_1mm_, PCT_2mm_, PCT_3mm_, PCT_4mm_).

Correlation of focal backscatter and pachymetry: A correlation analysis of the focal backscatter values (as an indicator for the presence of a fibrillar layer) with the corresponding pachymetry values was performed at the corneal apex and for 1 mm, 2 mm, 3 mm and 4 mm radial distance from the apex.

### 2.3. Statistics

Continuous variables were summarized by mean ± standard deviation (SD), categorical/nominal variables by counts and percentages. Group comparison of FL-positive vs. FL-negative eyes were performed using a 2-sided t-test or Pearson χ^2^-test, depending on the distribution. Pearson correlation coefficients were calculated (r = 0 to 1 positive correlation, r = 0 no correlation, r = 0 to −1 negative correlation; r ≥ 0.1 corresponds to a weak correlation, r ≥ 0.3 corresponds to a moderate correlation, r ≥ 0.5 corresponds to a strong correlation.). A *p* value of < 0.05 was considered statistically significant. No adjustments for multiple testing were made.

Statistical analysis and preparation of graphics were performed using statistical software packages (SPSS 27, IBM Corp., Armonk, NY, USA; creation of statistical charts using Prism 6, GraphPad software, San Diego, CA, USA).

## 3. Results

A total of *n* = 96 patients (*n* = 96 eyes) with advanced FECD were included in the study. The demographic details of all patients are shown in Table 1. Comparing the FL-positive and FL-negative groups, there were no significant differences in age, gender, lens status and time of Scheimpflug imaging. However, there were significant differences with regard to the modified Krachmer grading, with a higher proportion of modified Krachmer grade 6 patients in the FL-positive group and a higher proportion of modified Krachmer grade 5 patients in the FL-negative group (Table 1).

### 3.1. En Face Scheimpflug Backscatter Data Analysis

An FL was detected in 74% (*n* = 71) of all patients, whereas 26% (*n* = 25) were FL-negative. All en face Scheimpflug backscatter images showed a significant increase in hyperreflectivity toward the periphery (>10 mm), and the FL was displayable in higher definition in the corneal center compared to the periphery as previously described (Figure 1) [10,15]. Scheimpflug backscatter analysis showed no values for few individual peripheral corneal measurements (Table 2). The dimensions of the FL (*n* = 71) were for the area 9.97 ± 5.13 mm², for the vertical caliper diameter 3.50 ± 0.93 mm, for the horizontal caliper diameter FL 4.09 ± 1.10 mm, and for the maximum caliper diameter 4.38 ± 1.07 mm.

### 3.2. Comparative Analysis of Focal Backscatter in FL-Positive and FL-Negative Eyes

Eyes with FL (*n* = 71) showed an ACB of 31.7 ± 7.7 GSU and eyes without FL (*n* = 25) showed an ACB of 23.1 ± 4.8 GSU (*p* < 0.001). The focal peripheral backscatter values for eyes with FL and without FL, respectively, were for PCB_1mm_ 27.9 ± 6.4 GSU and 20.9 ± 3.5 GSU (*p* < 0.001), for PCB_2mm_ 23.4 ± 5.7 GSU and 19.2 ± 3.7 GSU (*p* = 0.001) for PCB_3mm_ 22.8 ± 5.5 GSU and 20.8 ± 4.9 GSU (*p* = 0.127) and for PCB_4mm_ 27.4 ± 6.6 GSU and 28.6 ± 10.1 GSU (*p* = 0.579).

The individual focal peripheral corneal backscatter values PCB_1mm_, PCB_2mm_, PCB_3mm_ and PCB_4mm_ at superior, nasal, inferior and temporal locations, respectively, are shown in Table 2. In FL-positive eyes, significantly increased GSU values at the corneal apex and at 1 mm radius, as well as inferiorly and temporally at 2 mm and 3 mm radius suggest a primarily central and inferotemporal localization of FL.

### 3.3. Pachymetry Data Analysis

Analysis of Scheimpflug pachymetry data in all FECD eyes (*n* = 96) showed a CCT of 604.3 ± 53.6 µm, an ACT of 603.9 ± 52.8 µm, a PCT_1mm_ of 606.5 ± 50.8 µm, a PCT_2mm_ of 618.4 ± 47.2 µm, a PCT_3mm_ of 646.9 ± 45.1 µm, and a PCT_4mm_ of 692.7 ± 49.6 µm.

### 3.4. Comparative Analysis of Pachymetry in FL-Positive vs. FL-Negative Eyes

Eyes with FL (*n* = 71) showed a CCT of 614.6 ± 51.8 µm and eyes without FL (*n* = 25) showed a CCT of 575.1 ± 48.4 µm (*p* = 0.001). In eyes with FL, the ACT was 614.0 ± 51.6 µm, and in eyes without FL, the ACT was 575.2 ± 46.2 µm (*p* = 0.001). Peripheral pachymetry values for FECD eyes with FL and without FL, respectively, were for PCT_1mm_ 615.7 ± 50.0 µm and 580.2 ± 44.2 µm (*p* = 0.002), for PCT_2mm_ 625.2 ± 47.6 µm and 599.1 ± 40.9 µm (*p* = 0.017) for PCT_3mm_ 651.0 ± 46.3 µm and 635.0 ± 39.9 µm (*p* = 0.128) and for PCT_4mm_ 695.1 ± 51.8 µm and 686.0 ± 43.2 (*p* = 0.435).

The individual peripheral corneal thickness values PCT_1mm_, PCT_2mm_, PCT_3mm_ and PCT_4mm_ at superior, nasal, inferior and temporal locations, respectively, are shown in Table 3. In FL-positive eyes, significantly increased pachymetry values at the corneal apex and 1 mm radius as well as inferiorly and temporally at 2 mm radius and temporally at 3 mm radius suggest a primarily central and inferotemporal localization of corneal edema.

### 3.5. Correlation Analysis

Correlation of fibrillar layer dimensions and pachymetry: Weak correlations were found between vertical caliper diameter and ACT (r =0.249, *p* = 0.036) and between vertical caliper diameter and PCT_1mm_ (r = 0.251, *p* = 0.035). There were no other significant correlations between the investigated FL dimensions and the pachymetry values.

Correlation of focal backscatter and pachymetry: Correlation analysis showed moderate correlation of ACB values and ACT values (r = 0.309, *p* = 0.002) and weak correlation of focal backscatter and pachymetric values at 1 mm (r = 0.269, *p* = 0.008) and 2 mm (r = 0.271, *p* = 0.007). There were no significant correlations between focal backscatter and pachymetry values at 3 mm (r = 0.146, *p* = 0.159) and 4 mm (r = 0.004, *p* = 0.973) radial distance from the apex.

## 4. Discussion

In the present study, Scheimpflug imaging demonstrates that FL detection is associated with increased corneal thickness and that the FL and increased corneal thickness are primarily located in the central and inferotemporal area of advanced FECD corneas.

Adding to the results from our previous studies showing significantly reduced endothelial cell density [9] and increased light scattering [10] in the FL area, this further supports a potential role of clinical FL detection as a criterion in progression assessment and therapeutic decision making in FECD patients in the future.

Keratoplasty represents the only established definite treatment option for FECD [2,16], and DMEK may be considered the therapeutic gold standard in advanced FECD disease. However, the quest for further minimization of surgical intervention and the limited global supply of donor tissue lead to the continuous development and optimization of the surgical and conservative therapeutic spectrum. New tissue-sparing modalities for the treatment of FECD include hemi- or quarter-DMEK, Descemet stripping only (DSO) or cultured corneal endothelial cell injection, as well as the combined or sole topical drug application including particularly rho-associated protein kinase (ROCK) inhibitors [17,18,19,20,21]. In addition, a customized DMEK based on Scheimpflug images of FL areas with pronounced endothelial loss may be an option to reduce tissue needed for transplantation and thereby potentially also immune reaction rates [16].

With the arrival of new therapeutic options, the optimization of clinical FECD classification systems becomes increasingly important for progression assessment but also for identification of the optimal timepoint, the optimal corneal endothelial area-to-treat and for the selection of the optimal therapeutic approach [22]. The development of such classifications requires a detailed understanding of the underlying pathology and its concomitant characteristic morphological alterations, which may be documented in vivo using a variety of clinical imaging techniques. In this context, previous histology studies demonstrated that subendothelial collagen deposits in the form of an FL are detectable in approximately 80% of patients with advanced FECD (modified Krachmer grades 5 or 6) [8], and that this FL marks areas of sharp and significant reduction in CEC density [9,10]. Moreover, comparing histology and clinical imaging, the FL was identified as a major source of light scattering, enabling FL visualization in vivo by en face Scheimpflug backscatter analysis [10].

Until now, it was unclear whether reduced CEC density in the FL area was accompanied by increased aqueous humor leakage into the corneal tissue and edema formation or whether the FL rather sealed the posterior cornea preventing the entry of aqueous humor in advanced FECD eyes [10]. Moreover, the detailed location of the FL at the posterior corneal surface was unknown.

Results of the present study demonstrate in vivo by Scheimpflug imaging that FL detection in advanced FECD eyes is associated with increased corneal thickness, suggesting edema formation. Moreover, it shows that the FL and increased corneal thickness are located in the central and inferotemporal region. The localization of edema in FL-positive eyes in the central and inferotemporal region replicate and complement previous findings by other groups also showing reduced endothelial cell count and confluent guttae, especially in the inferotemporal corneal area [23,24,25]. This supports the hypothesis that increased aqueous humor entry occurs in the FL area due to significant focal reduction in CEC density or even due to complete defects of significant size within the central and inferotemporal corneal endothelial compound. Furthermore, these results suggest that as a consequence, minimally invasive procedures in (FL-positive) patients with advanced FECD should particularly target the central and inferotemporal endothelium.

A weak correlation of the FL area dimensions (vertical caliper diameter) and corneal thickness was identified in our cohort, pointing toward a potential area growth of the fibrillar layer with FECD progression. However, the correlation was weak and likely limited by the restricted spectrum of FECD stages (exclusively modified Krachmer stages 5 and 6) investigated in the present study. Future studies will examine all stages of FECD in a larger cohort and will provide further insight into the development and area growth of the FL with FECD progression.

Early ex vivo studies of FECD histology specimens showed that FL thickness increases with stromal and epithelial edema (maximum FL thickness in this histologic study 9.3 µm) [26]. It was hypothesized that the increased influx of aqueous humor entering Descemet’s membrane through a rarified endothelium in decompensated corneas contributes to the focal formation of a loose FL continuously growing in thickness [26]. This suggests that the increase in corneal thickness of FL-positive eyes in our study is mainly attributable to corneal edema formation but, to a lesser extent, also to the FL itself. However, based on our experience, Scheimpflug imaging is so far insufficient in resolution to measure the thickness of the FL and thus replicate these studies in vivo. Recent investigations by other groups measuring the thickness of the endothelium/Descemet membrane complex provide evidence that comparable in vivo studies will be possible in the future [27,28]. Using high-definition-optical coherence tomography (HD-OCT) 3D thickness maps, these studies showed a strong correlation between endothelium/Descemet membrane thickness values and FECD severity [27].

Backscatter of the anterior (120 µm), mid and posterior (60 µm) cornea increased with FECD progression and thus with increase in corneal thickness in earlier studies [5,29,30]. In addition, posterior backscatter was among the variables selected by a statistical learning algorithm to predict edema resolution after DMEK in FECD patients based on preoperative assessment [31]. Moreover, posterior backscatter contributed particularly to the development of visual acuity-related disability [4], and there was an early drop in posterior backscatter following DMEK surgery in FECD patients, whereas anterior backscatter regressed over a longer period [15]. Our data confirm the original results from these studies and emphasize the significance of the FL as a source and as an important morphological correlate of posterior backscatter associated with increased edema in FECD. A comparison of patients with and without FL in similar study settings would be interesting to further investigate the role of the FL (and of its removal) as a morphologically relevant target structure in advanced FECD disease. It seems tempting to speculate that DMEK in patients with FL produces a similar backscatter-attenuating effect as a capsulotomy in posterior capsule opacification both through removing the most relevant source of light scattering.

The most important limitation of our study is the restricted range of FECD stages. Moreover, Scheimpflug backscatter images were not adjusted for variations in light intensity in our study. Such adjustments involve the acquisition of a standard scatter source before each examination with subsequent adjustment of the measurements in the human eye. Although these adjustments significantly improve the accuracy, they are not part of the standard automated Scheimpflug imaging procedure [29]. In accordance with previous studies showing reduced repeatability and reproducibility of densitometry in peripherally located corneal areas [32,33], Scheimpflug backscatter analysis showed no values for isolated peripheral corneal measurements in our study. However, the demonstration of a correlation between Scheimpflug FL detection and corneal thickness in our study suggests that the procedure may still be applicable with limitations in clinical practice as described herein.

## 5. Conclusions

A significant reduction in endothelial cell density, increased backscatter (both demonstrated in previous studies [9,10]), and increased corneal thickness are important features of the FL area, which may primarily be found in a central and inferotemporal location in advanced FECD eyes. This may provide important information for progression assessment and therapeutic decision making in FECD patients in the future. Furthermore, it may open avenues for customized surgical approaches based on preclinical Scheimpflug imaging.

## Figures and Tables

**Table 1:** Characteristics of patients and investigated eyes (one eye per patient), fibrillar layer (FL). FECD: Fuchs endothelial corneal dystrophy; AM: Ante Meridiem (before noon).

	FL Positive	FL Negative	Total	*p*-Value
Patients, *n* (%)	71 (74)	25 (26)	96 (100)	-
FECD Krachmer Grade 5	12 (16.9%)	13 (52%)	25 (26%)	<0.01
FECD Krachmer Grade 6	59 (83.1%)	12 (48%)	71 (74%)	
Age (years), mean ± standard deviation	67.2 ± 9.5	69.2 ± 9.2	67.7 ± 9.4	0.37
Female, *n* (%)	39 (54.9%)	13 (52%)	52 (54.2%)	0.80
Pseudophakic, *n* (%)	24 (33.8%)	5 (20.0%)	29 (30.2%)	0.20
Time of Scheimpflug imaging: AM, *n* (%)	22 (31%)	8 (32%)	30 (31.3%)	0.93

Data are numbers (percentage) or mean ± standard deviation.

**Table 2:** Focal backscatter in fibrillar layer (FL)-positive and -negative advanced FECD eyes: The en face Scheimpflug backscatter plane with the highest backscatter gray scale unit (GSU) at the corneal apex within the posterior corneal 100 µm was selected, and focal backscatter values were extracted at the given locations. Apex corneal backscatter (ACB), peripheral corneal backscatter (PCB).

Distance	Position	FL Positive	FL Negative	*p*-Value
		(*n* = 71)	(*n* = 25)	
ACB (GSU)	mean	31.7 ± 7.7	23.1 ± 4.8	*p* < 0.001
PCB_1mm_ (GSU)	mean	27.9 ± 6.4	20.9 ± 3.5	*p* < 0.001
	superior	24.4 ± 8.2	20.2 ± 4.0	*p* = 0.001
	nasal	25.4 ± 7.6	20.6 ± 3.7	*p* < 0.001
	inferior	30.0 ± 7.9	20.9 ± 4.4	*p* < 0.001
	temporal	31.4 ± 9.1	21.8 ± 3.4	*p* < 0.001
PCB_2mm_ (GSU)	mean	23.4 ± 5.7	19.2 ± 3.7	*p* = 0.001
	superior	22.0 ± 5.8	20.0 ± 5.1	*p* = 0.124
	nasal	21.7 ± 6.5	20.1 ± 5.2	*p* = 0.284
	inferior	23.9 ± 7.5	17.8 ± 3.5	*p* < 0.001
	temporal	25.9 ± 8.0	19.0 ± 3.3	*p* < 0.001
PCB_3mm_ (GSU)	mean ^1^	22.8 ± 5.5	20.8 ± 4.9	*p* = 0.127
	superior ^2^	28.3 ± 10.7	25.4 ± 8.4	*p* = 0.235
	nasal	23.2 ± 6.0	23.5 ± 9.5	*p* = 0.862
	inferior	19.4 ± 5.2	17.6 ± 2.9	*p* = 0.040
	temporal	20.0 ± 5.2	18.0 ± 3.9	*p* = 0.048
PCB_4mm_ (GSU)	mean ^3^	27.4 ± 6.6	28.6 ± 10.1	*p* = 0.579
	superior ^4^	40.4 ± 15.1	38.6 ± 23.6	*p* = 0.672
	nasal	34.1 ± 10.4	36.4 ± 12.4	*p* = 0.374
	inferior	20.6 ± 5.0	21.7 ± 5.4	*p* = 0.341
	temporal	19.9 ± 5.1	19.0 ± 4.2	*p* = 0.430

^1,2^*n* = 95 (FL pos. = 71; FL neg. = 24), ^3,4^ *n* = 83 (FL pos. = 60; FL neg. = 23).

**Table 3:** Corneal thickness in fibrillar layer (FL)-positive and -negative advanced FECD eyes. Apex corneal thickness (ACT), peripheral corneal thickness (PCT).

Distance	Position	FL Positive	FL Negative	*p*-Value
		(*n* = 71)	(*n* = 25)	
ACT (µm)	mean	614.0 ± 51.6	575.2 ± 46.2	*p* = 0.001
PCT_1mm_ (µm)	mean	615.7 ± 50.0	580.2 ± 44.2	*p* = 0.002
	superior	612.9 ± 51,0	583.9 ± 39.1	*p* = 0.011
	nasal	610.5 ± 49	581.8 ± 42.6	*p* = 0.011
	inferior	619.4 ± 50.6	579.0 ± 50.5	*p* = 0.001
	temporal	618.8 ± 54.0	576.2 ± 47.8	*p* = 0.001
PCT_2mm_ (µm)	mean	625.2 ± 47.6	599.1 ± 40.9	*p* = 0.017
	superior	625.9 ± 48.2	609.9 ± 38.1	*p* = 0.135
	nasal	620.9 ± 46.6	602.7 ± 39,5	*p* = 0.084
	inferior	627.4 ± 49.0	594.5 ± 46.5	*p* = 0.004
	temporal	626.5 ± 54.6	589.2 ± 47.7	*p* = 0.003
PCT_3mm_ (µm)	mean	651.0 ± 46.3	635.0 ±39.9	*p* = 0.128
	superior	659.8 ± 47.5	654.7 ± 42.4	*p* = 0.635
	nasal	654.3 ± 46.6	643.2 ± 41.2	*p* = 0.294
	inferior	645.7 ± 49.8	624.1 ± 41.8	*p* = 0.055
	temporal	644.4 ± 53.5	618.2 ± 44.1	*p* = 0.031
PCT_4mm_ (µm)	mean	695.1 ± 51.8	686.0 ± 43.2	*p* = 0.435
	superior	712.9 ± 56.5	713.8 ± 53.8	*p* = 0.939
	nasal	704.7 ± 53.7	696.6 ± 48.8	*p* = 0.507
	inferior	685.6 ± 61.2	672.6 ± 45.7	*p* = 0.333
	temporal	677.1 ± 56.1	661.0 ± 39.2	*p* = 0.189

## Data Availability

The data presented in this study are available on request from the corresponding author. The data are not publicly available due to privacy and ethical considerations.
